# Clinical implication of minimal residual disease assessment by next-generation sequencing-based immunoglobulin clonality assay in pediatric B-acute lymphoblastic leukemia

**DOI:** 10.3389/fonc.2022.957743

**Published:** 2022-09-15

**Authors:** Jae Wook Lee, Yonggoo Kim, Ari Ahn, Jong Mi Lee, Jae Won Yoo, Seongkoo Kim, Bin Cho, Nack-Gyun Chung, Myungshin Kim

**Affiliations:** ^1^ Department of Pediatrics, College of Medicine, The Catholic University of Korea, Seoul, South Korea; ^2^ Catholic Hematology Hospital, College of Medicine, The Catholic University of Korea, Seoul, South Korea; ^3^ Department of Laboratory Medicine, College of Medicine, The Catholic University of Korea, Seoul, South Korea; ^4^ Catholic Genetic Laboratory Center, Seoul St. Mary’s Hospital, College of Medicine, The Catholic University of Korea, Seoul, South Korea

**Keywords:** minimal residual disease (MRD), B-acute lymphoblastic leukemia (B-ALL), immunoglobulin clonality, next-generation sequencing (NGS), normalization

## Abstract

Measuring minimal residual disease (MRD) during treatment is valuable to identify acute lymphoblastic leukemia (ALL) patients who require intensified treatment to avert relapse. We performed the next-generation sequencing (NGS)-based immunoglobulin gene (Ig) clonality assay and evaluated its clinical implication in pediatric B-ALL patients to assess MRD. Fifty-five patients who were diagnosed and treated with *de novo* (*n* = 44) or relapsed/refractory B-ALL (*n* = 11) were enrolled. MRD assessment was performed using the LymphoTrack^®^ Dx IGH and IGK assay panels. The percentage of the clonal sequences per total read count was calculated as MRD (% of B cells). The data were normalized as the proportion of total nucleated cells (TNC) by LymphoQuant™ Internal control or the B-cell proportion in each sample estimated by flow cytometry or immunohistochemistry. Clonal Ig rearrangement was identified in all patients. The normalized MRD value was significantly lower than the unnormalized MRD value (*p* < 0.001). When categorizing patients, 27 of 50 patients (54%) achieved normalized MRD <0.01%, while 6 of them did not achieve MRD <0.01% when applying the unnormalized value. The normalized post-induction MRD value of 0.01% proved to be a significant threshold value for both 3-year event-free survival (100% for MRD <0.01% vs. 60.9% ± 10.2% for MRD ≥0.01%, *p* = 0.007) and 3-year overall survival (100% for MRD <0.01% vs. 78.3% ± 8.6% for MRD ≥0.01%, *p* = 0.011). However, unnormalized MRD was not a significant factor for outcome in this cohort. Our study demonstrated that MRD assessment by NGS-based Ig clonality assay could be applied in most pediatric B-ALL patients. Normalized post-induction MRD <0.01% was a significant prognostic indicator.

## Introduction

In pediatric acute lymphoblastic leukemia (ALL), event-free survival (EFS) rate has improved through the accurate identification of prognostic factors, the designation of risk group based on these factors, and treatment of appropriate duration and intensity according to risk group, done within the setting of cooperative clinical trials (1). Measuring minimal residual disease (MRD) during treatment is an additional risk factor to identify patients who require intensified treatment to avert relapse. Recently, it has been shown that the presence and the degree of MRD at specific time points during therapy can be used to guide treatment, demonstrating the clinical significance of detecting MRD (2, 3). Methods of evaluating MRD by reverse transcriptase qPCR (RT-qPCR), quantitative polymerase chain reaction (qPCR), multi-parametric flow cytometry (MFC), and next-generation sequencing (NGS)-based immunoglobulin (Ig) clonality assay have been shown to be promising MRD monitoring tools for B-ALL.

Specifically, NGS-based Ig clonality assay showed excellent analytical performance with high sensitivity and applicability to most B-cell neoplasia (4, 5). The most recent National Comprehensive Cancer Network^®^ recommended that a validated MRD assessment technology should have a sensitivity of at least 10^−4^ (6). In addition to the analytical performance including sensitivity, standardization is the other issue that should be addressed before clinical implication. MRD value could be reported differently according to the method: expression ratio of fusion gene per reference gene for RT-qPCR, patient-specific clonal gene burden calculated by standard curve for qPCR, and % of bone marrow (BM) mononuclear cells or total nucleated cells (TNCs) for MFC. NGS-based Ig clonality assay provides two values: % of B cell, which is calculated by clonal Ig read count per total Ig read count, and % of TNC, which is adjusted according to the proportion of B cells in each sample. However, it remains unclear which value is optimal for risk stratification in each patient and how clinical laboratories should determine the % of TNC when undergoing MRD assessment by NGS-based Ig clonality assay.

In this study, we performed the NGS-based Ig clonality assay and evaluated its clinical implication in pediatric B-ALL patients to assess MRD. We further clarified the method of normalization to calculate the clonal burden of % of B cells into % of TNC and elucidated the significance of both MRD values when applied to clinical decision-making.

## Materials and methods

### Patients and therapy

This study was approved by the institutional review board of Seoul St. Mary’s Hospital, which is affiliated with The Catholic University of Korea (IRB No: KC17TESI0187). Study participants were patients diagnosed with *de novo* or relapsed/refractory ALL at our institution from June 2016 to December 2018. Overall, 55 patients were enrolled: *de novo* ALL (*n* = 44), BM relapse (*n* = 10), and refractory (*n* = 1) (**Table 1**). One patient was considered refractory due to lack of response after 2 courses of remission induction chemotherapy. Diagnosis of ALL was based on BM pathology, immunophenotyping, cytogenetics, and molecular genetics, as shown in the World Health Organization (WHO) Classification of Tumours of Haematopoietic and Lymphoid Tissues (7). Recurrent genetic abnormalities were diagnosed according to previously reported methods (8). For the 44 *de novo* ALL patients, initial risk group classification was done according to our institutional regimen (9), and patients were classified as follows: low risk (*n* = 9, 20%), standard risk (*n* = 11, 25%), high risk (*n* = 11, 25%), and very high risk (*n* = 13, 30%). For the 10 relapsed patients, the median time from diagnosis to relapse was 40.5 months (range: 12.1–68.9 months).

**Table 1:** Patient characteristics.

	*n* = 55 (%)
Median age at diagnosis (range)	7.2 years (2.3–17.0)
Median initial WBC count (range)	19.70 × 10^9^/L (1.29–207.34)
**Disease status**	
*De novo*	44 (80)
Relapsed	10 (18)
Refractory	1 (2)
**Genetics**	
High hyperdiploidy	10 (18)
*ETV6-RUNX1*	8 (15)
*E2A-PBX1*	4 (7)
*BCR-ABL1*	2 (4)
Normal	18 (33)
Others	13 (24)

WBC, white blood cell count.

### Patient treatment and time point of MRD monitoring

The 44 *de novo* ALL patients were classified and treated according to an institutional protocol, the details of which have been previously reported (9). Forty-two patients achieved complete remission (CR) after remission induction chemotherapy, while two patients achieved delayed CR after additional chemotherapy. All except one patient were treated with chemotherapy only, while the remaining patient received allogeneic hematopoietic stem cell transplantation (HSCT) in the first CR due to molecular relapse prior to the delayed intensification phase of chemotherapy.

For the 11 relapsed/refractory patients, the reinduction regimens were as follows: vincristine, steroid, asparaginase, and anthracycline (daunorubicin or idarubicin) (four drug regimens, *n* = 6); four drug regimens with imatinib (*n* = 1); vincristine, steroid, and imatinib (*n* = 1); fludarabine, cytarabine, and idarubicin (*n* = 2); clofarabine, cyclophosphamide, and etoposide (*n* = 1). Ten patients achieved CR with reinduction chemotherapy, and these patients proceeded to allogeneic HSCT.

Samples were retrospectively retrieved for MRD assessment at the time of diagnosis, after induction [4 weeks, time point 1 (TP1)], consolidation (14 weeks, TP2), and 24–25 weeks (TP3). Patients who relapsed during follow-up were evaluated for Ig rearrangement again. MRD assessment was done in 50, 40, and 22 patients at TP1, TP2, and TP3, respectively, depending on the availability of samples for MRD analysis (**Supplementary Table 1**).

### MRD monitoring using NGS

Genomic DNA (gDNA) was isolated from BM aspirates using the QIAamp DNA minikit (Qiagen, Hilden, Germany). Samples were quantified using Qubit dsDNA BR assay (Thermo Fisher Scientific, Waltham, MA, USA). The LymphoTrack^®^ IGH FR1/2/3 and LymphoTrack^®^ IGK assay panels (InVivoScribe Technologies, San Diego, CA, USA) were used for the analysis of initial samples to determine clonal rearrangements and MRD samples to detect previously characterized clonotypic rearrangements. For MRD testing, low-positive controls were also included in every run.

All experiments were performed according to the manufacturer’s guidelines, which were previously reported (10). Briefly, amplification by PCR was performed using 240 ng of gDNA per sample, and master mixes contain primers designed with barcoded sequence adaptors. Next, we purified the amplicons using an Agencourt^®^ AMPure XP system (Beckman Coulter, Brea, CA, USA) and quantified the amplicons with a Qubit^®^ dsDNA HS Assay Kits (Thermo Fisher Scientific), High Sensitivity D1000 Reagents, and High Sensitivity D1000 ScreenTape (Agilent Technologies, Santa Clara, CA). The libraries were sequenced on a MiSeqDx instrument (Illumina, San Diego, CA, USA) using the MiSeq Reagent Kit version 2 (500 cycles), aiming at 500,000 reads per sample. We prepared one replicated libraries from the gDNA sample to analyze MRD; each library had 240 ng of gDNA input. Percentage confidence for which the searched sequence was not detected was 97.17% at 10^−4^.

The FASTQ files were analyzed using the LymphoTrack MRD software v2.0.2 (InVivoScribe Technologies) for clonality assessment and sequence tracking. Clonal rearrangement was determined according to the manufacturer’s guidelines. When the total number of reads for each sample was ≥20,000 and the top merged sequence had ≥2.5% of the total reads or when the total number of reads for each sample was ≥10,000 but <20,000 and the top merged sequence had ≥5% of the total reads, these results were interpreted as clonal. For MRD assessment, the clone of exact sequence matches and similar sequences (up to two mismatched nucleotides) were sought after chemotherapy according to the manufacturer’s guideline. If any sequences exact or similar to the initial clone were found, the amount of residual Ig clone was described as the proportion per total Ig read counts (% of B cell). All clonal rearrangements found at diagnosis in each patient were evaluated in subsequent MRD samples.

We tried to estimate the MRD clone in each sample by normalization using the following methods. LymphoQuant™ Internal control was added to each PCR reaction at 100 cell equivalency when testing these follow-up samples to allow the estimation of cell equivalents within each sample. The proportion of the MRD clone in each sample was calculated as % of TNC using the formulas provided by the manufacturer. Alternatively, we estimated the CD19-positive B-cell proportion in each sample using flow cytometry [FACSCanto II Flow Cytometer and FACSDiva software (Becton Dickinson, San Jose, CA, USA)] or immunohistochemical stain (IHC, mouse monoclonal anti-human CD19 antibody; NovoCastra, Newcastle upon Tyne, UK). The interchangeability among the methods for normalization has been evaluated in advance. The CD19-positive B-cell proportion analyzed by flow cytometry and IHC showed good correlation (*R* ^2^ = 0.9518, *p* < 0.001) and could be used interchangeably (**Supplementary Figure 1A**). In addition, we compared the MRD results that were normalized by LymphoQuant™ Internal control with those normalized by flow cytometry or IHC and found that they showed good correlation (*R* ^2^ = 0.8558, *p* < 0.001) (**Supplementary Figure 1B**).

The percentage of TNC was calculated using the following formula: (% of B-cell) × (CD19-positive B-cell proportion in sample)/100. For convenience, we annotated % of B cell and % of TNC as unnormalized and normalized MRD, respectively.

### Statistics and outcome measures

Event-free survival (EFS) was defined as time from diagnosis of ALL to last follow-up in CR, or first event. Relapse, early death, primary refractory disease, death in CR, and secondary malignancy were considered events. Patients with primary refractory disease or those who died during remission induction chemotherapy were considered to have events at time zero. For the relapsed patients, EFS was defined as time from relapse to last follow-up in CR, or subsequent event. Overall survival (OS) was defined as time from diagnosis (or relapse for the 10 relapsed patients) to last follow-up, or death from any cause. Comparison of EFS in the *de novo* cohort was done for the following variables: age (<10 years old vs. ≥10 years old), initial white blood cell (WBC) count (<50 × 10^9^/L vs. ≥50 × 10^9^/L), prephase steroid response during remission induction chemotherapy, presence of good prognosis genetic abnormalities (high hyperdiploidy or *ETV6-RUNX1*), and MRD at TP1 (<0.01% vs. ≥0.01%). Probabilities of EFS and OS were calculated using the Kaplan–Meier method, and comparison of survival curves according to risk factors was done with the log-rank test. Comparison of end of induction MRD value (negative vs. positive with a threshold of 0.01% normalized value) according to patient disease status (*de novo vs*. relapsed/refractory) was done with chi-square test. Patient follow-up was done up till 30 June 2021. Comparison between normalized and unnormalized MRD was performed by Wilcoxon signed-rank test, and their correlation was done by Spearman’s rho correlation. *p*-value <0.05 was considered significant.

## Results

Clonal Ig rearrangement was identified in all patients. *IGH* FR1 was useful in most patients (*n* = 49), and *IGH* FR2 and *IGK* were useful in three patients each. Twenty-four patients had one Ig clone and 20 patients had two. The other 11 patients showed more than three Ig clones. *IGH* V3-J4 rearrangement was most common followed by V3-J6 and V3-J5 (**Supplementary Figure 2**). The mean proportion of Ig clone at diagnosis was 54.153% ± 22.859%. During MRD assessments, we derived two MRD values: unnormalized (% of B cell) and normalized MRD (% of TNC). These two MRD values showed good correlation with a correlation coefficient of 0.968 (*p* < 0.001). The average and standard deviation (SD) of unnormalized MRD was 10.397% ± 23.253%, 1.311% ± 3.196%, and 1.535% ± 3.557% at TP1, TP2, and TP3, respectively. The normalized MRD value was significantly lower than unnormalized MRD (*p* < 0.001). The average and SD of normalized MRD was 2.649% ± 10.545% at TP1, 0.059% ± 0.173% at TP2, and 0.058% ± 0.189% at TP3.

Then, we categorized patients according to the MRD value 0.01%, 0.1%, and 1%. We observed that there was a difference between before and after normalization. At TP1, 27 of 50 patients (54%) achieved normalized MRD <0.01%, while 21 (42%) showed unnormalized MRD <0.01%. At TP2, 34 of 40 patients (85%) showed normalized MRD <0.01% while 29 (72.5%) showed unnormalized MRD <0.01%. At TP3, 16 (73%) and 15 (68%) of 22 patients showed normalized and unnormalized MRD <0.01, respectively. Overall, 12 patients were recategorized from MRD ≥0.01% to MRD <0.01% after normalization. Considering that therapy adjustment decisions may be made based on MRD <0.01% threshold at TP1, the results of six patients indicated the need for more intensified treatment due to MRD ≥0.01% prior to normalization (**Supplementary Figure 3**).

Events in the *de novo* cohort of patients included eight patients who relapsed at a median of 22.4 months from diagnosis (range: 17.1–47.6 months). Two patients died of relapsed/refractory disease. The estimated 3-year EFS and OS of the *de novo* cohort was 88.6% ± 4.8% (36/44) and 95.3% ± 3.2% (42/44), respectively. All 11 patients followed from the point of relapsed/refractory ALL achieved subsequent CR. However, 6 of the 11 patients experienced further events: subsequent relapse (*n* = 5) and secondary malignancy (*n* = 1). Overall, four patients died: three from relapsed/refractory disease and one from acute respiratory distress syndrome in CR. The 3-year EFS and OS of the relapsed/refractory cohort were 45.5% ± 15.0% (5/11) and 63.6% ± 14.5% (7/11), respectively. Utilizing a normalized MRD threshold of 0.01%, 26 of 40 *de novo* ALL patients (65%) with evaluable data were TP1 MRD negative, while only 1 of 10 relapsed/refractory patients (10%) were TP1 MRD negative (**Table 2**, *p* = 0.003 when comparing the two patient groups).

**Table 2:** Correlation between patient disease status and end of induction minimal residual disease value using a threshold value of 0.01.

		End of induction normalized MRD value		Total
		<0.01%	≥0.01%	
Disease status	*De novo*	26	14	40
	Relapsed/refractory	1	9	10
	Total	27	23	50

MRD, minimal residual disease.

**Figure 1:**
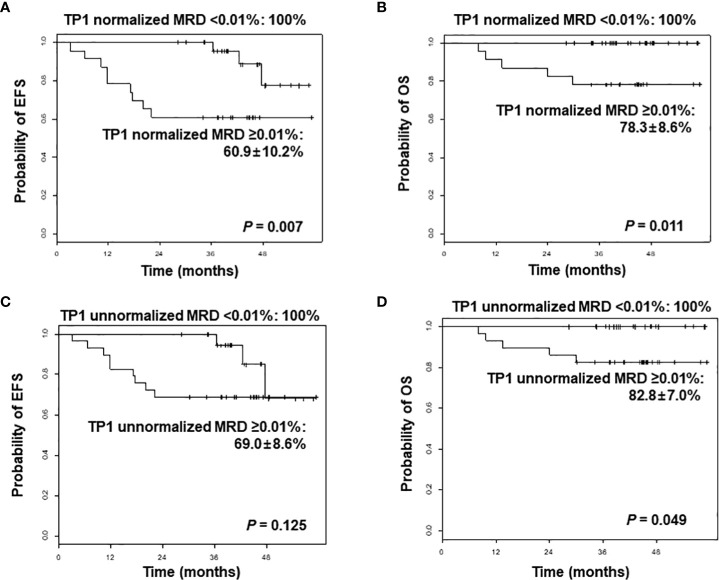
Comparison of event-free survival (EFS) and overall survival (OS) of overall patients according to normalized minimal residual disease (MRD) values **(A, B)**, and unnormalized MRD values **(C, D)** after induction, analyzed by next-generation sequencing-based immunoglobulin clonality assay. TP1, time point 1.

In combining the *de novo* and relapsed/refractory ALL cohorts, normalized TP1 MRD value of 0.01% proved to be a significant threshold value for both 3-year EFS (100% for MRD <0.01% vs. 60.9% ± 10.2% for MRD ≥0.01%, *p* = 0.007) and 3-year OS (100% for MRD <0.01% vs. 78.3 ± 8.6% for MRD ≥0.01%, *p* = 0.011). However, unnormalized TP1 MRD was not a significant factor for EFS in this cohort (3-year EFS 100% for MRD <0.01% vs. 69.0 ± 8.6% for MRD ≥0.01%, *p* = 0.125) (**Figures 1A–D**). When limiting the analysis to the *de novo* ALL cohort, the initial WBC count proved to be a significant factor for EFS: 3-year EFS of 96.7% ± 3.3% (initial WBC count <50 × 10^9^/L) vs. 71.4% ± 12.1% (initial WBC count ≥50 × 10^9^/L), *p* = 0.027. Patients with a normalized TP1 MRD <0.01% had superior outcome compared with those with MRD ≥0.01%, although without statistical significance (3-year EFS 100% vs. 78.6% ± 11.0%, *p* = 0.229).

## Discussion

The prognostic significance of MRD, measured from the BM at specific time points after therapy, is well-established. Basically, cellular MRD counts have general prognostic value at the cutoff level of 0.01% MRD cells (10^−4^), indicating 1 in 10,000 cells in a specimen. Because MRD values are reported in various ways according to the assessment technology, standardization is essential to establish the strategy for monitoring patients. In terms of NGS-based Ig clonality assay, data normalization and the quality control (QC) of robust amplification, library preparation, and sequencing are technically important. Several procedures were established to address the normalization issue such as a central in-tube QC spiked to each tube as library control and calibrator and central polytartget QC (11, 12). The LymphoQuant™ internal controls are used for in-tube QC and are probed to be optimal for normalization, showing better correlation with the MFC results (**Supplementary Figure 4**).

In this study, we could identify clonal Ig rearrangement in all pediatric B-ALL patients by an NGS-based Ig clonality assay. A total of 89% of cases were successfully characterized using FR1 primer sets, similar to the results of previous studies in B-cell neoplasia (4, 5). The frequency of common V-J rearrangements was also in line with our previous study (10). Unnormalized and normalized MRD values showed good correlation, which was predicted because both values were calculated based on clonal Ig read count. The values, however, differed with regard to prognostic relevance. The most significant factor resulting in difference was the proportion of B cells in each sample. At diagnosis, most cells were B cells with clonal Ig rearrangement. After treatment, normal hematopoietic components of erythroid and granulocytic lineages were reconstructed, leading to a relatively lower B-cell proportion. Accordingly, total Ig read count was low in those samples even though the amount of input DNA was sufficient, resulting in a relatively high unnormalized MRD value. For example, we found that six TP1 MRD-negative patients were categorized as having persistent MRD before normalization. More importantly, unnormalized TP1 MRD did not predict patient outcome whereas the normalized TP1 MRD was a significant prognostic factor for both EFS and OS. Therefore, normalization is a pivotal process for MRD assessment to remain an efficient prognostic indicator and a factor in therapy modification in ALL, as well as to prevent chemotherapy intensification of limited value (13). Post-therapy MRD should be able to indicate prognosis in *de novo* ALL patients. A clear limitation of our results was that for the 44 patients with *de novo* ALL, patients with normalized TP1 MRD <0.01% had higher EFS than those with MRD ≥0.01%, but without statistical significance. Initial WBC count was the only significant factor for EFS, with the threshold WBC count set at 50 × 10^9^/L as defined in the National Cancer Institute/Rome criteria for high-risk ALL (14). At present, we are implementing NGS-based MRD measurement in all of our ALL patients, and a subsequent, larger-scale study may clarify the role of MRD at TP1 using this modality in determining patient outcome.

The important prognostic role of end of induction MRD detected in the BM has been established through both flow cytometry and PCR detection of Ig and T-cell receptor gene rearrangements (15, 16). NGS-based Ig clonality assay is likely more sensitive than previous methods of MRD detection, and may also be able to predict patients with worse outcome. One recent study comparing NGS-based MRD assessment and flow cytometry with a threshold of 0.01% found that NGS identified 38.7% more patients as MRD positive (17). Importantly, these patients had significantly lower EFS than those who were MRD negative according to NGS, indicating overall that NGS had a lower false-negative rate than flow cytometry. Further studies are necessary to determine the prognostic role of MRD assessment using an NGS-based Ig assay, as well as the optimum threshold for risk group classification.

Consequently, our study demonstrated that MRD assessment by NGS-based Ig clonality assay could be applied in most pediatric B-ALL patients. TP1 MRD with a threshold of 0.01% could be a valid prognostic factor. Importantly, normalization of MRD measurements as % of TNC using LymphoQuant internal control or the B-cell proportion in the sample allowed for NGS-based MRD to become a significant prognostic indicator.

## Data availability statement

The original contributions presented in the study are included in the Supplementary Material, further inquiries can be directed to the corresponding author.

## Ethics statement 

This study was reviewed and approved by Institutional review board of Seoul St. Mary’s Hospital(IRB No: KC17TESI0187). Written informed consent to participate in this study was provided by the participants’ legal guardian/next of kin.

## Author contributions

YK, N-GC, and MK were responsible for the study concept and project administration. JWL, AA, JML, JWY, SK, BC, and N-GC acquired resources. JWL, AA, and MK analyzed and interpreted data. JWL, YK, N-GC, and MK wrote the manuscript. All authors contributed to the article and approved the submitted version.

## Funding

This research was supported by a grant from the Korea Health Technology R&D Project through the Korea Health Industry Development Institute (KHIDI), funded by the Ministry of Health and Welfare, Republic of Korea (grant number: HI18C0480).

## Acknowledgments

The authors wish to thank the Catholic Genetic Laboratory Center for their contribution to the experiments.

## Conflict of interest

The authors declare that the research was conducted in the absence of any commercial or financial relationships that could be construed as a potential conflict of interest.

## Publisher’s note

All claims expressed in this article are solely those of the authors and do not necessarily represent those of their affiliated organizations, or those of the publisher, the editors and the reviewers. Any product that may be evaluated in this article, or claim that may be made by its manufacturer, is not guaranteed or endorsed by the publisher.

## References

[1] Lee JW Cho B. Prognostic factors and treatment of pediatric acute lymphoblastic leukemia. Korean J Pediatr (2017) 60(5):129–37. doi: 10.3345/kjp.2017.60.5.129 PMC5461276 28592975

[2] Campana D Pui CH. Minimal residual disease-guided therapy in childhood acute lymphoblastic leukemia. Blood (2017) 129(14):1913–8. doi: 10.1182/blood-2016-12-725804 PMC5383866 28167658

[3] Pui CH Pei D Raimondi SC Coustan-Smith E Jeha S Cheng C. Clinical impact of minimal residual disease in children with different subtypes of acute lymphoblastic leukemia treated with response-adapted therapy. Leukemia (2017) 31(2):333–9. doi: 10.1038/leu.2016.234 PMC5288281 27560110

[4] Arcila ME Yu W Syed M Kim H Maciag L Yao J. Establishment of immunoglobulin heavy (IGH) chain clonality testing by next-generation sequencing for routine characterization of b-cell and plasma cell neoplasms. J Mol Diagn (2019) 21(2):330–42. doi: 10.1016/j.jmoldx.2018.10.008 PMC6436112 30590126

[5] Ho C Syed M Roshal M Petrova-Drus K Moung C Yao J. Routine evaluation of minimal residual disease in myeloma using next-generation sequencing clonality testing: Feasibility, challenges, and direct comparison with high-sensitivity flow cytometry. J Mol Diagn (2021) 23(2):181–99. doi: 10.1016/j.jmoldx.2020.10.015 PMC7874334 33217553

[6] Network NCC. Pediatric acute lymphoblastic leukemia (Version 1.2022) (2022). Available at: https://www.nccn.org/professionals/physician_gls/pdf/ped_all.pdf.

[7] Borowitz MJ CJ Downing JR Le Beau MM. B-lymphoblastic leukaemia/lymphoma with recurrent genetic abnormalities. In: Swerdlow SHCE Harris NL Jaffe ES Pileri SA Stein H Thiele J, editors. WHO classification of tumours of haematopoietic and lymphoid tissues, vol. . p . Lyon: IARC (2017). p. 203–9.

[8] Lee JW Kim Y Cho B Kim S Jang PS Lee J. High incidence of RAS pathway mutations among sentinel genetic lesions of Korean pediatric BCR-ABL1-like acute lymphoblastic leukemia. Cancer Med (2020) 9(13):4632–9. doi: 10.1002/cam4.3099 PMC7333828 32378810

[9] Lee JW Kim SK Jang PS Jeong DC Chung NG Cho B. Treatment of children with acute lymphoblastic leukemia with risk group based intensification and omission of cranial irradiation: A Korean study of 295 patients. Pediatr Blood Cancer (2016) 63(11):1966–73. doi: 10.1002/pbc.26136 27463364

[10] Jo I Chung NG Lee S Kwon A Kim J Choi H. Considerations for monitoring minimal residual disease using immunoglobulin clonality in patients with precursor b-cell lymphoblastic leukemia. Clin Chim Acta (2019) 488:81–9. doi: 10.1016/j.cca.2018.10.037 30389459

[11] Brüggemann M Kotrová M Knecht H Bartram J Boudjogrha M Bystry V. Standardized next-generation sequencing of immunoglobulin and T-cell receptor gene recombinations for MRD marker identification in acute lymphoblastic leukaemia; a EuroClonality-NGS validation study. Leukemia (2019) 33(9):2241–53. doi: 10.1038/s41375-019-0496-7 PMC6756028 31243313

[12] Knecht H Reigl T Kotrová M Appelt F Stewart P Bystry V. Quality control and quantification in IG/TR next-generation sequencing marker identification: Protocols and bioinformatic functionalities by EuroClonality-NGS. Leukemia (2019) 33(9):2254–65. doi: 10.1038/s41375-019-0499-4 PMC6756032 31227779

[13] Kruse A Abdel-Azim N Kim HN Ruan Y Phan V Ogana H. Minimal residual disease detection in acute lymphoblastic leukemia. Int J Mol Sci (2020) 21(3):1054. doi: 10.3390/ijms21031054 PMC7037356 32033444

[14] Smith M Arthur D Camitta B Carroll AJ Crist W Gaynon P. Uniform approach to risk classification and treatment assignment for children with acute lymphoblastic leukemia. J Clin Oncol (1996) 14(1):18–24. doi: 10.1200/jco.1996.14.1.18 8558195

[15] Borowitz MJ Devidas M Hunger SP Bowman WP Carroll AJ Carroll WL. Clinical significance of minimal residual disease in childhood acute lymphoblastic leukemia and its relationship to other prognostic factors: a children's oncology group study. Blood (2008) 111(12):5477–85. doi: 10.1182/blood-2008-01-132837 PMC2424148 18388178

[16] Conter V Bartram CR Valsecchi MG Schrauder A Panzer-Grümayer R Möricke A. Molecular response to treatment redefines all prognostic factors in children and adolescents with b-cell precursor acute lymphoblastic leukemia: Results in 3184 patients of the AIEOP-BFM ALL 2000 study. Blood (2010) 115(16):3206–14. doi: 10.1182/blood-2009-10-248146 20154213

[17] Wood B Wu D Crossley B Dai Y Williamson D Gawad C. Measurable residual disease detection by high-throughput sequencing improves risk stratification for pediatric b-ALL. Blood (2018) 131(12):1350–9. doi: 10.1182/blood-2017-09-806521 PMC5865233 29284596

