# Supplementary material for: Clinical implication of minimal residual disease assessment by next-generation sequencing-based immunoglobulin clonality assay in pediatric B-acute lymphoblastic leukemia
Source: Front Oncol. 2022 Sep 15;12:957743. doi: 10.3389/fonc.2022.957743 (PMC9521036; doi:10.3389/fonc.2022.957743)
Supplement: Supplementary file 1 [file DataSheet_1.docx]

Supplementary Material

Clinical Implication of Minimal Residual Disease Assessment by Next-Generation Sequencing-Based Immunoglobulin Clonality Assay in Pediatric B-Acute Lymphoblastic Leukemia


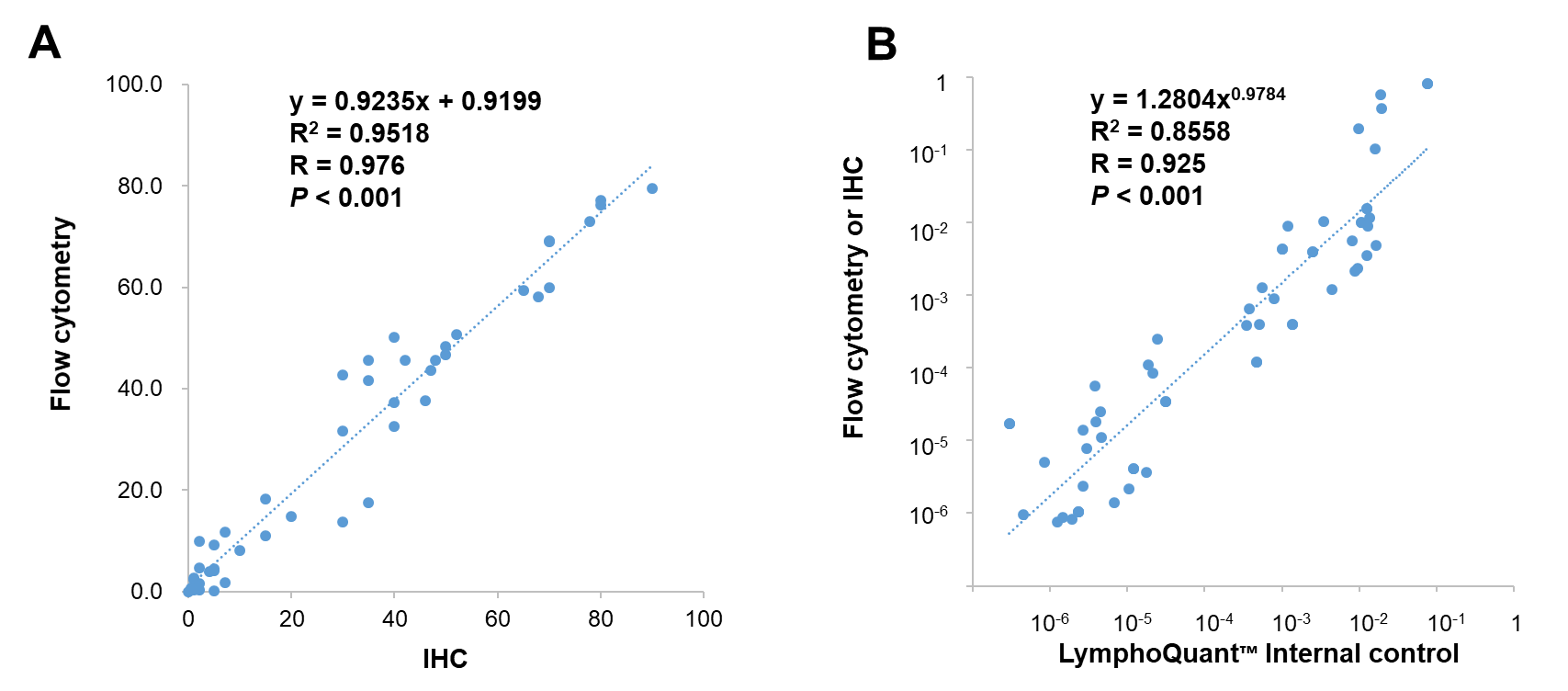


Supplementary Figure 1. (A) Correlation of CD19-positive B-cell proportion which was analyzed by flow cytometry and immunocytochemistry (IHC). (B) Correlation of minimal residual disease value normalized by LymphoQuant™ Internal control and flow cytometry or IHC.

Supplementary Figure 2. Frequency of V-J rearrangements in all cohort.

**Supplementary Figure 3.** Distribution of minimal residual disease (MRD) category before and after normalization.

**
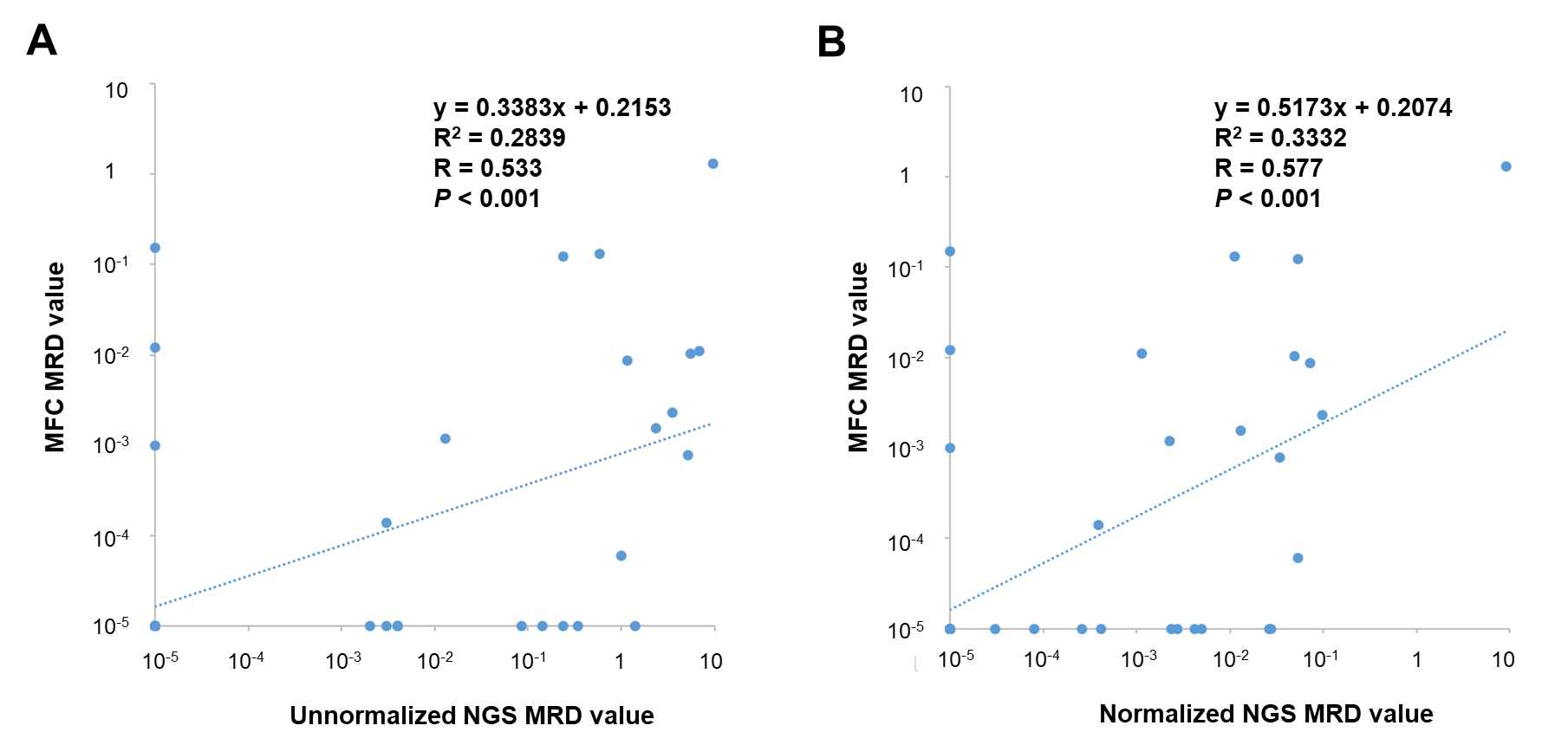
**

**Supplementary Figure 4.** Comparison minimal residual disease (MRD) value analyzed by next-generation sequencing (NGS)-based immunoglobulin gene assay and multiparametric flow cytometry (MFC). Normalized MRD value (A) showed better correlation with MFC than unnormalized MRD value (B). MFC was performed using antibodies CD22-PC7 (Beckman Coulter), CD66c (Beckman Coulter) + CD123-PE (Miltenyi Biotech), and CD73 (BD Biosciences) + CD304 (Biolegend)-APC. In total, up to 1×10^5^ cells were acquired using DxFLEX flow cytometers (Beckman Coulter), and all acquired data files were analyzed using the Kaluza software v2.1 (Beckman Coulter) according to our gating strategy

**Supplementary Table 1.** Minimal residual disease changes according to immunoglobulin clones

| No | Age at Dx | Disease status | FR type | Dx | Initial | FU1 | FU2 | FU3 |
| --- | --- | --- | --- | --- | --- | --- | --- | --- |
| 1 | 3.7 | de novo | FR1 | B-ALL, NOS | O▲X | – | – | – |
| 2 | 8.0 | de novo | FR2 | B-ALL, NOS | O | O | O | O |
| 3 | 10.2 | de novo | FR1 | B-ALL, NOS | O▲ | O▲ | O▲ | O▲ |
| 4 | 9.8 | de novo | FR1 | B-ALL, NOS | O▲ | – |  |  |
| 5 | 8.7 | de novo | FR1 | B-ALL, NOS | O | O | – | – |
| 6 | 4.4 | de novo | FR1 | B-ALL with hyperdiploidy | O▲X■ | – | – | – |
| 7 | 3.7 | de novo | FR1 | B-ALL with hyperdiploidy | O▲ | – |  |  |
| 8 | 2.6 | de novo | IGK | B-ALL, NOS | O | O | O |  |
| 9 | 9.7 | BM relapse | FR1 | B-ALL, NOS | O▲ | O▲ |  |  |
| 10 | 3.3 | de novo | FR1 | B-ALL  with t(12;21)(p13.2;q22.1); *ETV6-RUNX1* | O | – |  |  |
| 11 | 6.1 | BM relapse | FR2 | B-ALL, NOS | O | O |  |  |
| 12 | 2.3 | de novo | FR1 | B-ALL with hyperdiploidy | O▲X | O▲X |  | – |
| 13 | 3.5 | de novo | FR2 | B-ALL, NOS | O |  | – |  |
| 14 | 3.1 | de novo | FR1 | B-ALL, NOS | O▲ | – | – |  |
| 15 | 15.5 | de novo | FR1 | B-ALL, NOS | O | O | – |  |
| 16 | 15.8 | de novo | FR1 | B-ALL, NOS | O | O | – | – |
| 17 | 12.7 | BM relapse | FR1 | B-ALL with t(9;22)(q34.1;q11.2); *BCR-ABL1* | O | O | – | O |
| 18 | 3.8 | BM relapse | FR1 | B-ALL, NOS | O▲ | O▲ |  |  |
| 19 | 3.0 | de novo | FR1 | B-ALL, NOS | O▲X | – |  |  |
| 20 | 2.7 | de novo | FR1 | B-ALL  with t(12;21)(p13.2;q22.1); *ETV6-RUNX1* | O▲ | O – | O – | – |
| 21 | 4.6 | de novo | FR1 | B-ALL, NOS | O | – |  |  |
| 22 | 14.4 | de novo | FR1 | B-ALL with  t(1;19)(q23;p13.3); *TCF3-PBX1* | O | O | – | – |
| 23 | 17.0 | de novo | FR1 | B-ALL, NOS | O | O | – | – |
| 24 | 4.3 | BM relapse | IGK | B-ALL, NOS | O▲X■ | – | – |  |
| 25 | 13.7 | BM relapse | FR1 | B-ALL, NOS | O▲ | O▲ | O▲ | – ▲ |
| 26 | 4.9 | BM relapse | FR1 | B-ALL, NOS | O | O | O |  |
| 27 | 3.7 | de novo | FR1 | B-ALL with hyperdiploidy | O▲ | – | – |  |
| 28 | 4.8 | BM relapse | FR1 | B-ALL, NOS | O▲X | O▲X |  |  |
| 29 | 12.0 | de novo | FR1 | B-ALL, NOS | O▲X■ | O▲X■ | O–X– | – |
| 30 | 11.7 | de novo | FR1 | B-ALL, NOS | O▲ | – |  |  |
| 31 | 13.8 | de novo | IGK | B-ALL, NOS | O▲ |  | – |  |
| 32 | 13.7 | de novo | FR1 | B-ALL with  t(1;19)(q23;p13.3); *TCF3-PBX1* | O▲ | – | – |  |
| 33 | 5.0 | de novo | FR1 | B-ALL  with t(12;21)(p13.2;q22.1); *ETV6-RUNX1* | O | – | – |  |
| 34 | 7.3 | de novo | FR1 | B-ALL with  t(1;19)(q23;p13.3); *TCF3-PBX1* | O | O | – | O |
| 35 | 4.1 | de novo | FR1 | B-ALL with hyperdiploidy | O▲X |  | – | – |
| 36 | 5.1 | de novo | FR1 | B-ALL  with t(12;21)(p13.2;q22.1); *ETV6-RUNX1* | O | – | – | – |
| 37 | 2.7 | de novo | FR1 | B-ALL with hyperdiploidy | O | O | – | – |
| 38 | 17.0 | de novo | FR1 | B-ALL, NOS | O▲X | O – X |  |  |
| 39 | 5.3 | de novo | FR1 | B-ALL  with t(12;21)(p13.2;q22.1); *ETV6-RUNX1* | O▲ | – ▲ | O▲ |  |
| 40 | 15.7 | de novo | FR1 | B-ALL, NOS | O | – |  |  |
| 41 | 9.2 | de novo | FR1 | B-ALL, NOS | O | O | O | O |
| 42 | 9.9 | de novo | FR1 | B-ALL, NOS | O▲ | – |  |  |
| 43 | 12.0 | BM relapse | FR1 | B-ALL with t(9;22)(q34.1;q11.2); *BCR-ABL1* | O▲X |  | – – X | – |
| 44 | 7.2 | de novo | FR1 | B-ALL with iAMP21 | O▲ | O – | – |  |
| 45 | 14.8 | de novo | FR1 | B-ALL, NOS | O |  | – | O |
| 46 | 16.6 | de novo | FR1 | B-ALL, NOS | O | O | – | O |
| 47 | 3.1 | de novo | FR1 | B-ALL, NOS | O | O | – |  |
| 48 | 3.5 | de novo | FR1 | B-ALL, NOS | O▲X | – | – |  |
| 49 | 5.7 | de novo | FR1 | B-ALL, NOS | O | – |  |  |
| 50 | 15.5 | de novo | FR1 | B-ALL, NOS | O▲ | O▲ | – |  |
| 51 | 12.3 | BM relapse | FR1 | B-ALL, NOS | O▲ | O▲ | O▲ |  |
| 52 | 15.8 | refractory | FR1 | B-ALL, NOS | O | O | O | O |
| 53 | 4.4 | de novo | FR1 | B-ALL  with t(12;21)(p13.2;q22.1); *ETV6-RUNX1* | O▲ | – | – |  |
| 54 | 2.3 | de novo | FR1 | B-ALL  with t(12;21)(p13.2;q22.1); *ETV6-RUNX1* | O▲ | – | – |  |
| 55 | 11.2 | de novo | FR1 | B-ALL with  t(1;19)(q23;p13.3); *TCF3-PBX1* | O▲ | – | – |  |

Dx, diagnosis; FU, follow-up; BM, bone marrow; B-ALL, B-acute lymphoblastic leukemia; NOS, not otherwise specified; O, clone 1; ▲, clone 2; X, clone 3; ■, clone 4; –, not detected
